# Vaccination with DNA Plasmids Expressing Gn Coupled to C3d or Alphavirus Replicons Expressing Gn Protects Mice against Rift Valley Fever Virus

**DOI:** 10.1371/journal.pntd.0000725

**Published:** 2010-06-22

**Authors:** Nitin Bhardwaj, Mark T. Heise, Ted M. Ross

**Affiliations:** 1 Department of Infectious Diseases and Microbiology, Graduate School of Public Health, University of Pittsburgh, Pittsburgh, Pennsylvania, United States of America; 2 Center for Vaccine Research, University of Pittsburgh, Pittsburgh, Pennsylvania, United States of America; 3 Department of Microbiology and Molecular Genetics, University of Pittsburgh, Pittsburgh, Pennsylvania, United States of America; 4 Department of Microbiology and Immunology, The Carolina Vaccine Institute, University of North Carolina, Chapel Hill, North Carolina, United States of America; Tulane School of Public Health and Tropical Medicine, United States of America

## Abstract

**Background:**

Rift Valley fever (RVF) is an arthropod-borne viral zoonosis. Rift Valley fever virus (RVFV) is an important biological threat with the potential to spread to new susceptible areas. In addition, it is a potential biowarfare agent.

**Methodology/Principal Findings:**

We developed two potential vaccines, DNA plasmids and alphavirus replicons, expressing the Gn glycoprotein of RVFV alone or fused to three copies of complement protein, C3d. Each vaccine was administered to mice in an all DNA, all replicon, or a DNA prime/replicon boost strategy and both the humoral and cellular responses were assessed. DNA plasmids expressing Gn-C3d and alphavirus replicons expressing Gn elicited high titer neutralizing antibodies that were similar to titers elicited by the live-attenuated MP12 virus. Mice vaccinated with an inactivated form of MP12 did elicit high titer antibodies, but these antibodies were unable to neutralize RVFV infection. However, only vaccine strategies incorporating alphavirus replicons elicited cellular responses to Gn. Both vaccines strategies completely prevented weight loss and morbidity and protected against lethal RVFV challenge. Passive transfer of antisera from vaccinated mice into naïve mice showed that both DNA plasmids expressing Gn-C3d and alphavirus replicons expressing Gn elicited antibodies that protected mice as well as sera from mice immunized with MP12.

**Conclusion/Significance:**

These results show that both DNA plasmids expressing Gn-C3d and alphavirus replicons expressing Gn administered alone or in a DNA prime/replicon boost strategy are effective RVFV vaccines. These vaccine strategies provide safer alternatives to using live-attenuated RVFV vaccines for human use.

## Author Summary

Rift Valley fever virus (RVFV) is an arthropod-borne phlebovirus associated with abortion storms, neonatal mortality in livestock and hemorrhagic fever or fatal encephalitis in a proportion of infected humans. Requirement of multiple booster immunizations to maintain the level of protective immunity with the inactivated vaccines and the ability of live-attenuated vaccines to cause detrimental side-effects are major limitations preventing the widespread use of current vaccines. In this paper, we describe the use of DNA and alphavirus replicon based vaccination approaches to elicit a protective immune response against RVFV. While both vaccines elicited high titer antibodies, DNA vaccination elicited high titer neutralizing antibodies, whereas the replicon vaccine elicited cellular immune responses. Both strategies alone or in combination elicited immune response that completely protected against not only mortality, but also illness. Even though the delivery vectors elicited some protection on their own, they did not prevent severe morbidity. These promising vaccines provide an alternative RVFV vaccine for livestock and humans.

## Introduction

Rift Valley fever (RVF) is an arthropod-borne viral zoonosis. The causative agent Rift Valley fever virus (RVFV) belongs to the genus *Phlebovirus* of the family *Bunyaviridae* and was first discovered in the Rift Valley of Kenya in 1931 [1]. RVFV infections in livestock are characterized by an acute hepatitis, abortion and high mortality rates, especially in new born or young animals. Human infection with RVFV typically leads to a mild flu-like febrile illness. However, ∼2% of infected individuals have more severe complications, such as retinal degeneration, fatal hepatitis, severe encephalitis and hemorrhagic fever [2]. The ability of RVFV to cross geographic or national boundaries, coupled with the fact that RVFV replicates in a wide range of mosquito vectors, have raised concerns that the virus might spread further into non-endemic regions of the world. Before 1977, RVFV circulation was not detected beyond the Sub-Saharan countries. However, since 1997, RVFV outbreaks have occurred in Egypt [3], Mauritania in 1987 and 1998 [4], Saudi Arabia and Yemen [5]. In 2006–2007, RVFV outbreaks were recorded in Kenya, Somalia and Tanzania that resulted in human infections and deaths [6]. Thus, the ability of RVFV to cause explosive “virgin soil” outbreaks in previously unaffected regions demonstrates the need for prophylactic measures for this significant veterinary and public health threat.

The virus genome is composed of three single-stranded negative-sense RNA segments. The large (L) segment (∼6.4kb) encodes for the RNA-dependent RNA polymerase [7]. A medium (M) segment (∼3.8kb) encodes for four known proteins in a single open reading frame (ORF). These include the two structural glycoproteins, Gn and Gc, and the 14kDa non-structural NSm protein and the 78kDa NSm-Gn fusion peptide [7], [8], [9]. The small (S) segment is ambisense and encodes for the 1.6kDa viral nucleoprotein (N) in genomic orientation, as well as a non-structural (NSs) protein in the anti-genomic orientation [7]. The nonstructural genes (NSs and NSm) function to suppress host antiviral responses [10], [11].

RVFV is an important zoonotic pathogen with the potential to emerge in new areas through the spread of infected insect vectors or livestock or though intentional release as a bioterror agent. [12]. Inactivated RVFV vaccine (TSI-GSD-200) have been shown to elicit protective immunity in humans [13], however multiple booster vaccinations are required to achieve protective immunity, and perhaps most importantly, for many individuals, immunity rapidly wanes in the absence of follow-up booster vaccinations [13]. A modified live virus vaccine, based upon the Smithburn strain, is available for livestock in Africa [14], but it can cause pathology, spontaneous abortions, and teratogenic effects [15], [16], furthermore, animals vaccinated with live attenuated RVFV strains cannot be differentiated from naturally infected livestock, which may preclude export of these animals to non-RVFV endemic areas. One vaccine candidate under evaluation for human use is MP12, which is a mutagen-attenuated strain of the Egyptian RVFV isolate, ZH548 [17]. This vaccine was developed for use in both humans and livestock, with encouraging results in initial animal trials, but may cause teratology in pregnant animals [18]. In addition to the adverse effects of the live-attenuated vaccines, there are considerable safety concerns including incomplete attenuation, reversion back to virulent form during the vaccine manufacturing process. Therefore, new approaches are necessary to develop safe and effective vaccines.

Given limitation of existing RVFV vaccines, there is a need to explore alternative vaccine approaches. Previous studies have shown that DNA vaccines can elicit protective anti-RVFV immunity. Studies from our group and others have demonstrated that the molecular adjuvant C3d can significantly enhance antibody responses against DNA vaccine delivered antigens [19], [20], [21], [22], [23], [24], [25]. C3d adjuvanticity involves C3d binding to the complement receptor 2 (CR2) that is located on the surface of follicular dendritic cells (FDC), B cells, and T cells in many species (For review, see Toapanta and Ross) [26]. C3d stimulates antigen presentation by FDCs and helps to maintain immunological B cell memory. On B cells, C3d interaction with CR2 collects molecules, such as CD19 and TAPA. CD19 has a long intracellular tail that triggers a signaling cascade that results in cell activation and proliferation. Furthermore, simultaneous C3d–CR2 ligation and surface immunoglobulin (sIg) by antigen, activates two signaling pathways that cross-talk and synergize to activate B cells, thereby leading to enhanced antibody secretions specifically directed to the fused antigen. Therefore, we assessed whether fusion of murine C3d to the RVFV Gn glycoprotein would result in enhanced RVFV specific immune responses in the context of DNA vaccination.

Alphavirus replicon vectors are single hit vectors capable of eliciting potent systemic and mucosal immune responses against a wide range of pathogens, including hemorrhagic fever viruses, such as Lassa and Ebola [27]. Recently, we and others have demonstrated that alphavirus replicons based upon either VEE or Sindbis viruses were capable of eliciting protective anti-RVFV immune responses when the vectors expressed both RVFV glycoproteins from the RVFV M segment [28], [29] However, there has not been a direct comparison between DNA and alphavirus-based vectors, or an assessment of whether combining these vaccine strategies results in enhanced immunity or qualitative differences in the RVFV specific immune response. Furthermore, to date, RVFV vaccination studies have focused on antibody responses, and the ability of different vaccination strategies to elicit RVFV specific T cell responses has not been evaluated. Therefore, studies were conducted to directly compare DNA vaccines expressing either Gn or Gn-C3d to alphavirus vectors expressing Gn, evaluate whether combining these vaccines in a DNA prime/replicon boost strategy provided any advantage over either vaccine on its own, and to assess the nature of the antibody and T cell response elicited by each of these vaccine strategies.

## Materials and Methods

### Plasmid DNA

pTR600, a eukaryotic expression vector, has been described previously [23]. Briefly, the vector was constructed to contain the cytomegalovirus immediate-early promoter (CMV-IE) plus intron A (IA) for initiating transcription of eukaryotic inserts and the bovine growth hormone polyadenylation signal (BGH poly A) for termination of transcription. The vector contains the Col E1 origin of replication for prokaryotic replication and the kanamycin resistance gene (Kan^r^) for selection in antibiotic media.

**Figure 1:**
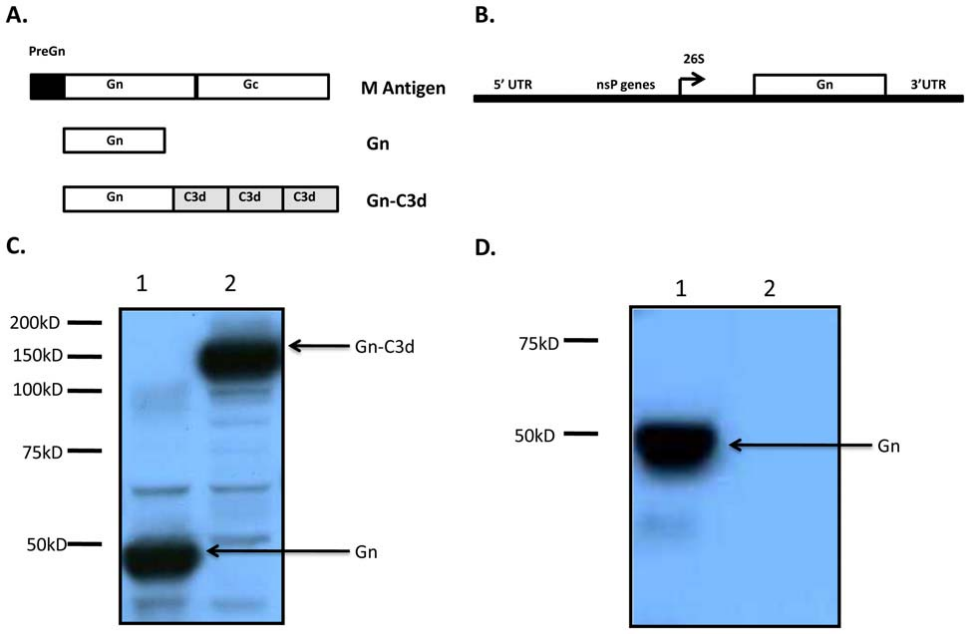
RVFV DNA and replicon vaccine expression constructs and analysis of expression of RVFV Gn from them. (**A**). Schematic representation of the entire polyprotein encoded my RVFV M segment (top) and the fragments (middle and bottom) cloned downstream of the tpA leader sequence of DNA vector. (**B**). Schematic representation of VEE replicon plasmid encoding Gn. (**C**). Proteins expressed from 293T cells transiently transfected with plasmid DNAs were assessed by SDS-PAGE and Western blot. The membrane was probed with anti-RVFV polyclonal antibody. (**D**). Proteins expressed from BHK21 cells infected with packaged VEE replicons were assessed by SDS-PAGE and Western blot. The membrane was probed with anti-RVFV polyclonal sera.

The gene sequence encoding for the RVFV, isolate ZH548 (Genbank DQ380206), Gn glycoprotein was used to PCR amplify a soluble form of Gn (Gn) without the transmembrane and cytoplasmic tail (Fig. 1A). The Gn gene sequence was cloned into the pTR600 vaccine vector by using unique *Hind*III *and Bam*HI restriction endonuclease sites. This Gn segment encoded a region from amino acids 131 to 557 (427 amino acids) and terminated in the sequence VAHCP. The vectors expressing Gn fused to three tandem repeats of the mouse homologue of C3d were cloned in frame and designated Gn-C3d, similar to constructs previously described [30]. Linkers composed of two repeats of four glycines and a serine [(G_4_S)_2_] were fused at the junctures of Gn and C3d and between each C3d repeat. Potential proteolytic cleavage sites between the junctions of Gn and the junction of C3d were mutated by ligating *Bam*HI and *Bgl*II restriction endonuclease sites to mutate an Arg codon to a Gly codon [30].

The plasmids were amplified in *Escherichia coli* DH5α; purified by using endotoxin-free, anion-exchange resin columns (Qiagen, Valencia, CA, USA); and stored at −20°C in distilled H_2_O. Plasmids were verified by appropriate restriction enzyme digestion and gel electrophoresis. Purity of DNA preparations was determined based on the optical density (O.D.) at wavelengths of 260 and 280 nm.

### Replicons

A soluble form of RVFV Gn lacking the transmembrane and cytoplasmic tail (see above) was introduced behind the 26S subgenomic promoter of the VEE replicon plasmid pVR21 as outlined in Figure 1B. VEE replicons expressing influenza hemagglutinin were used as negative controls. VEE replicon plasmids, as well as capsid and glycoprotein plasmids were linearized with NotI, replicon and helper transcripts were generated using mMessage mMachine T7 transcription kits (Ambion), and transcripts electroporated into BHK-21 cells to package replicon particles as described previously [31]. Following packaging, the replicons underwent two rounds of safety testing to ensure that no detectable replication competent virus was present [29], [31] at which point the replicons were concentrated by ultracentrifugation through a 20% sucrose cushion and titered using polyclonal antiserum against the VEE nonstructural proteins. Expression of the truncated RVFV Gn protein from the replicon was confirmed by western blot with a Gn specific monoclonal antibody (RV5 3G2-1A) generously provided by Dr. George Ludwig, USAMRIID, Ft. Detrick, Frederick, MD, USA.

### *In vitro* expression of vaccine plasmids

The human embryonic kidney cell line, 293T, was transfected (at 5×10^5^ cells/transfection) with 5µg of DNA by using Lipofectamine 2000 (Invitrogen, Carlsbad, CA, USA.) according to the manufacturer's guidelines. Supernatants were collected and stored at −20°C. Cell lysates were collected in 500µl of 1% Triton X-100 buffer and stored at −20°C.

To detect specific proteins in the cell supernatant, 1.5% of supernatant was diluted 1∶2 in SDS sample buffer (Bio-Rad, Hercules, CA, USA) and loaded onto a 10% polyacrylamide–SDS gel. The resolved proteins were transferred onto a nitrocellulose membrane (Bio-Rad, Hercules, CA, USA) and incubated with a 1∶5,000 dilution of anti-RVFV mouse sera in phosphate-buffered saline (PBS) containing 0.05% Tween 20 and 5% skim milk powder. After an extensive washing, bound mouse antibodies were detected by using a 1∶5,000 dilution of horseradish peroxidase-conjugated goat anti-mouse antiserum and enhanced chemiluminescence (Amersham, Buckinghamshire, United Kingdom).

### Animals and Immunizations

Six-to-eight week old female BALB/c mice (Harlan Sprague-Dawley, Indianapolis, IN, USA) were used for inoculations. Mice, housed with free access to food and water, were cared for under U.S. Department of Agriculture guidelines for laboratory animals. Mice were anesthetized with 0.03 to 0.04ml of a mixture of 5ml of ketamine HCl (100 mg/ml) and 1ml of xylazine (20 mg/ml). Gene gun immunizations were performed on shaved abdominal skin by using the hand-held Bio-Rad gene delivery system as described previously [23], [32], [33], [34]. For DNA immunizations, mice were immunized with three times at three week intervals with 2µg of DNA per 0.5mg of approximately 1-µm gold beads (Bio-Rad, Hercules, CA, USA) at a helium pressure setting of 400 lb/in^2^. For replicon immunizations mice were given one dose at week 6 or three doses at weeks 0, 3, and 6 of 1×10^5^ infectious unit (IU) of replicons by foot pad route. Blood samples were collected at at weeks 0, 2, 5, and 8 post-vaccination. A schematic of the vaccine regimen is listed in Table 1. Use of animals in this study was reviewed and approved by the University of Pittsburgh Institutional Animal Care and Use Committee (IACUC).

**Table 1:** Vaccine groups and vaccination regimen.

Immunogens	Immunization schedule			Route
	week 0	week 3	week 6	
Gn	Gn	Gn	Gn	GG
Gn-C3d	Gn-C3d	Gn-C3d	Gn-C3d	GG
Rep-Gn	Rep-Gn	Rep-Gn	Rep-Gn	FP
Gn-C3d/Rep-Gn	Gn-C3d	Gn-C3d	Rep-Gn	GG/FP
MP12.wk0	MP12	-	-	IP
MP12.wk6	-	-	MP12	IP
WIV MP12	WIV MP12	WIV MP12	WIV MP12	IP
DNA control	DNA control	DNA control	DNA control	GG
Rep control	Rep control	Rep control	Rep control	FP
Naives	-	-	-	-

GG (gene gun), FP (foot pad) and IP (intraperitoneal).

### Live attenuated and whole inactivated virus vaccines

The attenuated strain RVFV MP12 (MP12) and ZH501 was propagated and titrated using Vero cells. A pre-titrated RVFV MP12 was inactivated with 1% beta-propiolactone to a final concentration of 0.1% to make a whole virus inactivated preparation (WIV MP12). To ensure complete inactivation, an aliquot of inactivated virus was used to infect Vero cells and verify the lack of cytopathic effect (data not shown). BALB/c mice (n = 5) received a single intraperioteneal injection (i.p.) of MP12 (1×10^5^ PFU) 2 weeks or 8 weeks prior to infection (Table 1). Another group of mice was administered (i.p.) 3 doses of the WIV MP12 vaccine (1×10^5^ PFU equivalent).

### Immunological assays

Endpoint ELISA was performed on collected serum samples to assess the anti-Gn immunoglobulin G (IgG) response. Briefly, plates were coated with 100µl of inactivated RVFV MP12 overnight at 4°C, blocked with 5% non-fat dry milk in PBS-T (1h) at 25°C, and then extensively washed with PBS-T. Serial dilutions of mouse antisera were allowed to bind (1h) and the plates thoroughly washed with PBS-T. Subsequently, the primary antisera were detected by anti-mouse IgG conjugated to horseradish peroxidase (Bio-Rad, Hercules, CA, USA). The reaction was detected using tetramethybenzidine (TMB) substrate (Sigma, Saint Louis, MO, USA) (1 h) at 25°C. IgG isotypes were also assessed by ELISA as previously described [23], [35]. The secondary antibodies specific for IgG_1_, IgG_2a_, IgG_2b_ and IgG_3_ (Southern Biotechnology, Birmingham, AL, USA) were used at varying concentrations determined by optimization.

### Neutralizing antibody assays

Antibody-mediated neutralization of RVFV ZH501 was measured using plaque reduction and neutralization test (PRNT) [36]. Briefly, 100 plaque-forming units (PFU)/0.1 ml of RVFV ZH501 was mixed with serial two fold dilutions of heat inactivated (60°C for 30 min) serum samples in 96-well tissue culture plates. Virus-serum mixtures were incubated at 4°C overnight and placed into duplicate 23-mm wells (0.1ml/well) containing confluent monolayers of Vero cells (2×10^5^). Cells were incubated for 1h at 37°C and 5% CO_2_ and overlaid with nutrient medium containing 0.8% agar, 5% fetal bovine serum, 200U penicillin/ml, and 200mg streptomycin/ml. The plates were incubated at 37°C and 5% CO_2_. After 4 days of incubation, cells were fixed with 10% formalin and stained with 1% crystal violet for visualization of plaques. The neutralizing antibody titer of a serum was considered positive at the highest initial serum dilution that inhibited >50% of the plaques as compared to the virus control titration.

### ELISPOT assays

The number of anti-Gn specific murine INF-γ (mINF-γ) secreting splenocytes was determined by enzyme-linked immunospot (ELISpot) assay (R & D Systems, Minneapolis, MN, USA). Briefly, pre-coated anti-mIFN-γ plates were incubated (25°C for 1h) with RPMI (200µL) supplemented with 10% fetal calf serum and then incubated with splenocytes (5×10^5^/well) isolated from vaccinated mice. Cells were stimulated (48h) with peptides (15mers overlapping by 11 amino acids) representing the ectodomain of Gn glycoprotein. IL-2 was added to all wells (10 units/ml). Control wells were stimulated with PMA (+) (50 ng)/ionomycin (500 ng) or were mock stimulated (−). Plates were washed with PBS-T (3×) and were incubated (37°C for 48h; 5% CO_2_) with biotinylated anti-mIFN-γ and incubated (4°C for 16h). The plates were washed and incubated (25°C for 2h) with strepavidin conjugated to alkaline phosphatase. Following extensive washing, cytokine/antibody complexes were incubated (25°C for 1h) with stable BCIP/NBT chromagen. The plates were rinsed with dH_2_O and air-dried (25°C for 2h). Spots were counted by an ImmunoSpot ELISpot reader (Cellular Technology Ltd., Cleveland, OH, USA).

### RVFV ZH501 challenge

At week 8 of the study, a challenge dose containing 1×10^3^ PFU of RVFV ZH501 were administered i.p. During challenge, mice were housed in sealed negative-ventilation bio-containment units (Allentown Inc., Allentown, NJ, USA). All manipulations with infected mice and/or samples involving RVFV ZH501 were performed under strict BSL-3 enhanced conditions. The animals were examined twice daily for visual signs of morbidity or mortality, using a lab validated scoring system as previously described [37]. Mice were observed for clinical signs that ranged from lethargy, ruffled fur, and weight loss to neurological manifestations, such as hind-limb paralysis. Mice found in a moribund condition were euthanized.

### Passive transfer of immune sera and RVFV challenge

Sera from vaccinated mice were diluted 1∶10 in sterile PBS and 100µl of the diluted sera was injected (i.p.) into new, naïve BALB/c mice. One hour following transfer, the mice were challenged (i.p.) with virulent RVFV ZH501 (1×10^3^ PFU). Mice were observed daily for 8 days post-transfer for signs of morbidity and mortality.

### Statistics

Differences in ELISA titers and virus neutralization titers between various vaccine groups were analyzed by one-way ANOVA, followed by Tukey's multiple comparison test. Analysis of results from sickness score and weight loss were assessed by two-way ANOVA tests followed by Bonferroni's post tests. Statistical results are represented in the figure by * (P<0.05), ** (P<0.01), *** (P<0.001). Statistical analyses were done using GraphPad Prism software.

## Results

### Anti-Gn antibody responses

**Figure 2:**
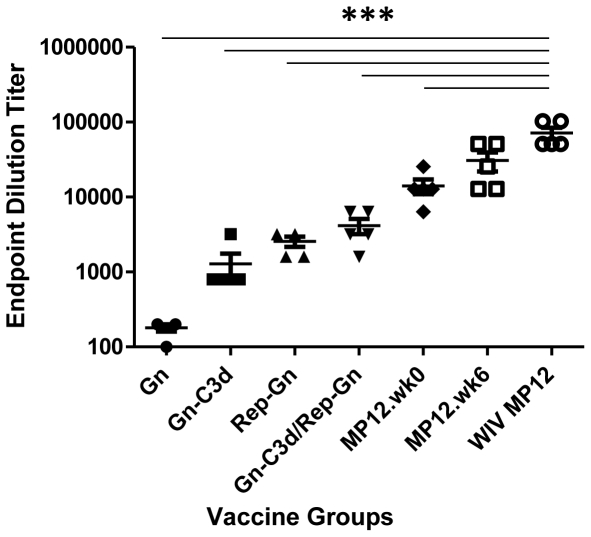
Indirect ELISA measuring RVFV specific IgG responses in mice immunized with indicated vaccine regimens. All groups received primary and two booster immunizations (except MP12) spaced 3 weeks apart. Serum samples were collected two weeks after the last immunization (week 8 of the study), except for group of mice vaccinated at week 0 with MP12 (MP12.wk0) which was challenged 8 weeks post-vaccination. End point dilution titers were conducted by diluting the sera until the OD values reached the background levels. Each dot represents an individual mouse. Error bars denote the standard error within the samples with a measurable titer. Representative data from 1 of 2 experiments shown. A 1-way ANOVA with Tukey's multiple comparison test was used to determine the significance of the data between groups, which is denoted by asterisks; *** p<0.001 for both MP12 vaccine regimens compared to the other vaccine regimens.

**Figure 3:**
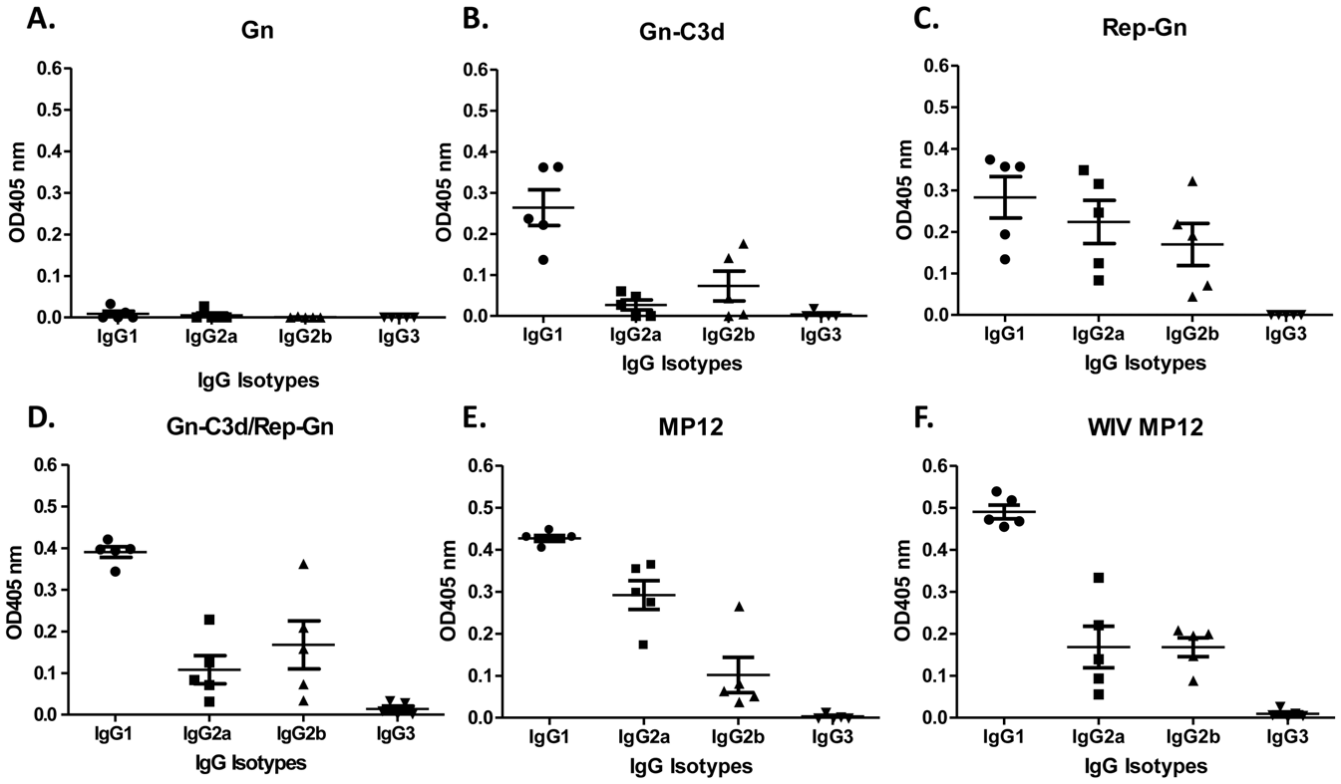
Isotype ELISA measuring RVFV specific IgG isotype responses in mice with indicated vaccine regimens from week 8 sera. 1∶100 dilution of serum samples from each vaccine group (**A**) to (**F**) were used and the results are represented in OD values. Each dot represents an individual mouse. Error bars denote the standard error within the samples with a measurable titer. Representative data from 1 of 2 experiments shown.

A truncated, soluble forms of Gn from the RVFV isolate ZH548 alone or fused to three copies of murine C3d (Gn-C3d) efficiently secreted from cells transfected with DNA plasmid and replicon vector (Fig. 1). RVFV Gn migrated at a 45kDa molecular weight and the C3d fusion with Gn increased the molecular weight to 135kDa. After 3 vaccinations, mice vaccinated with DNA plasmid expressing Gn elicited anti-Gn antibodies (1∶180), however, the fusion of C3d to Gn enhanced the anti-Gn antibodies (1∶1280), while mice vaccinated with replicons expressing Gn (Rep-Gn) had an average anti-Gn titer of 1∶2560 (Fig. 2). There were no detectable antibodies following a single DNA vaccination (data not shown). In order to determine if Gn-C3d-DNA could prime and enhance antibody titers following a Rep-Gn boost, mice were vaccinated twice with Gn-C3d-DNA and then administered a single inoculation of replicon expressing Gn. These vaccinated mice had higher anti-Gn antibody titers (1∶4160) compared to mice vaccinated with a single vaccination of alphavirus-replicon (1∶280). Mice vaccinated the Gn-DNA only, did not elicit any detectable anti-Gn antibodies (Fig. 3A). These antibody responses were comparable to mice immunized with live attenuated RVFV (MP12), but 1–2 logs lower than mice vaccinated with three doses of whole-inactivated RVFV (WIV MP12). MP12 infection elicited a mixed Th_1_ and Th_2_ response, whereas mice vaccinated with three doses of WIV MP12 had a Th_2_-restricted immune response (Fig. 3E and F). Mice vaccinated with Gn-C3d-DNA vaccines elicited predominately IgG_1_, suggesting a Th_2_ immune response (Fig. 3B and D). In contrast, the replicons expressing Gn administered to mice three times elicited not only IgG_1_, but also IgG_2a_ and IgG_2b_ isotypes suggesting a mixed Th_1_/Th_2_ response similar to that elicited by the live attenuated MP12 vaccine (Fig. 3C). Interestingly, mice primed with Gn-C3d-DNA maintained an IgG_1_ isotype bias following a boost with Gn expressing replicons (Fig. 3D). These titers were specific to the Gn antigen, since controls (DNA plasmid with no insert and replicons expressing the influenza virus hemagglutinin) did not elicit anti-Gn antibodies (data not shown).

### Elicitation of antibodies that neutralize virus infection

**Figure 4:**
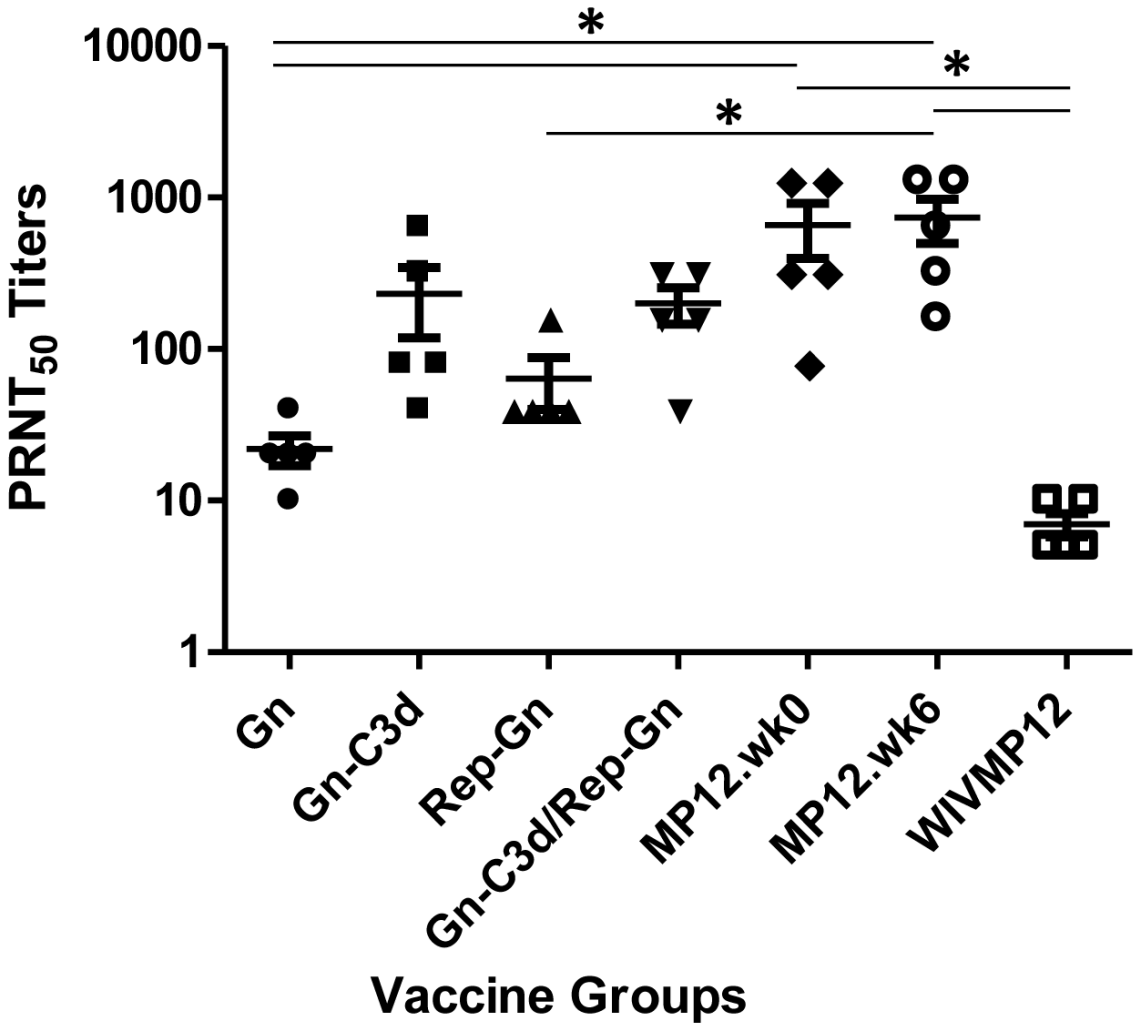
Neutralizing antibody responses of mice vaccinated with indicated vaccine regimens. PRNT_50_ titers of week 8 sera from mice immunized with indicated vaccines. Each dot represents an individual mouse. Error bars denote the standard error within the samples with a measurable titer. A 1-way ANOVA with Tukey's multiple comparison test was used to determine the significance of the data between groups, which is denoted by asterisks; * P<0.05.

At week 8 of the study, sera from mice vaccinated Gn-C3d-DNA orRep-Gn neutralized (PRNT_50_) RVFV ZH501, while priming mice with Gn-C3d-DNA and then boosting with Rep-Gn did not significantly enhance the neutralizing titers compared to Gn-C3d-DNA or Rep-Gn alone (Fig. 4). Mice vaccinated with the live attenuated MP12 vaccine strain had the highest neutralizing titers (average; 1∶656–1∶736) regardless if the mice were vaccinated at week 0 or week 6 of the study, and they were significantly higher than sera from mice vaccinated with Gn, Rep-Gn and WIV MP12 (p<0.05). In contrast, serum samples collected from Gn (1∶22) vaccinated or WIV MP12 (1∶8) had low virus neutralizing titers in spite of the fact that WIV MP12 elicited very high RVFV specific antibody levels as measured by ELISA (Figure 2).

### Elicitation of cellular immune responses

Mice vaccinated with DNA and replicon vaccines were challenged with MP12 virus two weeks after last immunization and splenocytes were collected 6 days post-infection. Cells collected from mice vaccinated with Gn vaccines were stimulated *in vitro* with 8 overlapping pools of peptide (15mers with overlapping by 11) specific for Gn. Mice vaccinated with Rep-Gn or Gn-C3d/Rep-Gn had responses to pools B and C (Table 2), representing a stretch of 111 amino acids starting at amino acid 53 in the Gn sequence. Only mice vaccinated with Gn-C3d/Rep-Gn had splenocyte responses to pool A. No responses were recorded from any mice to pools D-G. A few spots (10–12 spots) were detected following stimulation of splenocytes with an irrelevant peptide or unstimulated following *in vitro* re-stimulation. Mice vaccinated with DNA vaccines did not elicit cellular responses (Table 2). In addition, no spots were detected above background from splenocytes collected from naïve mice immunized with MP12 at day 6 post-infection (data not shown).

**Table 2:** Anti-Gn cell mediated immune responses of mice vaccinated with indicated vaccine regimens.

Pools	A	B	C	D	E	F	G	H
**Gn**	0	0	0	0	0	0	0	0
**Gn-C3d**	0	0	0	0	0	0	0	0
**Rep-Gn**	0	**541**	**415**	0	0	0	0	0
**Gn-C3d/Rep-Gn**	**77**	**301**	**253**	0	0	0	0	0
**Mock/Rep-Gn**	**0**	**171**	**124**	0	0	0	0	0
**Naive**	0	0	0	0	0	0	0	0

A group of mice vaccinated with different vaccine regimens were challenged with MP12 virus at week 8 of the study and 6 days post-infection splenocytes were isolated and stimulated with overlapping RVFV Gn specific peptides (pools A to H). Each pool contains 13 overlapping peptides except pool H which contains 14. Responses are represented as average number of spots (SFU per million cells) from different vaccine groups.

**Figure 5:**
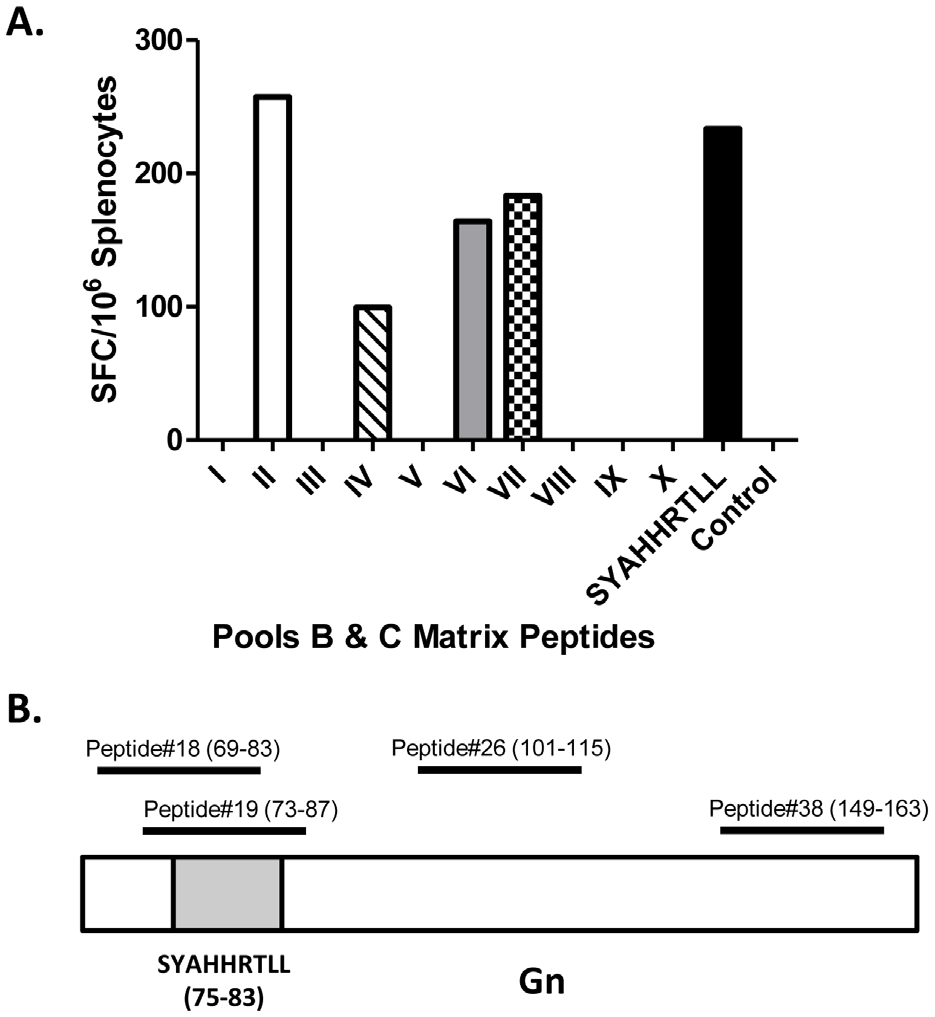
Identification of the peptide sequence eliciting cellular immune response in mice vaccinated with replicons. (**A**). Mice immunized with Rep-Gn vaccine were challenged with MP12 virus at week 8 of the study and 6 days post-infection splenocytes were isolated and stimulated with overlapping RVFV Gn specific peptides representing pools B and C and peptide SYAHHRTLL. Responses are represented as average number of spots (SFU per million cells). The highlighted peptides 18 and 19 share a common amino acid sequence SYAHHRTLL. Representative data from 1 of 2 experiments shown. (**B**). Schematic alignment of identified peptides with Gn. Numbers in the parentheses represent amino acid positions of the individual peptide. The gray box indicates the region of Gn covered by the predicted CD8+ T cells epitope SYAHHRTLL.

The peptides in these pools B and C were further analyzed to determine the peptides responsible to eliciting these responses in replicon-vaccinated mice. Using a matrix format, 4 out of 10 pools (5 peptides/pool) were identified (peptide pools II, IV, VI, VII) (Fig. 5A). From this analysis, four potential peptides (peptide # 18, 19, 36, 38) were identified as responsible for the vaccine elicited cellular responses. Two out of four peptides share a common amino acid sequence (SYAHHRTLL) predicted to be MHC class I restricted (www.immuneepitope.org). A unique peptide representing this region of Gn elicited similar mINF-γ cellular immune response as the four individual peptides as indicated in Figure 5B.

### DNA and replicon vaccines protect mice against virulent virus challenge

**Figure 6:**
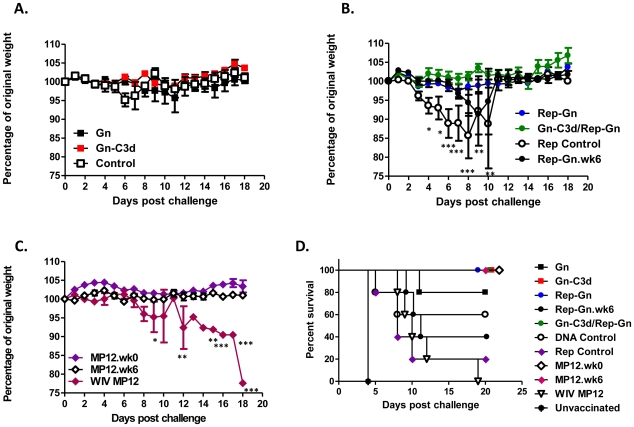
Weight loss curves and survival against virulent RVFV challenge of mice vaccinated with indicated vaccine regimens. Mice vaccinated with indicated vaccines or appropriate controls, DNA plasmid with no insert (DNA control) and replicon expressing influenza HA (replicon control) were challenged with 1000 PFU of RVFV ZH501 and monitored for loss in body weight (**A**) to (**C**) and mortality (**D**) daily post-challenge. Dead and moribund mice were included in the weight loss curves on the day of death, but not after. The daily weight of each mouse was compared to her weight on the day of challenge, and data are shown as the average percentage of initial weight for each cohort. Error bars represent the standard error for all samples available at that time point. A two-way ANOVA with Bonferroni's post tests was used to determine the significance of the body weight data between groups, which is denoted by asterisks; *P<0.05, **P<0.01, *** P<0.001. All vaccinated mice showed statistically significant protection (P<0.05, log rank test) compared to unvaccinated mice.

**Figure 7:**
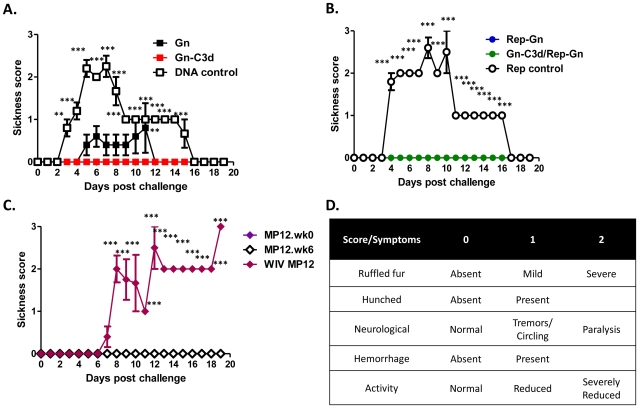
Post-challenge sickness score in mice vaccinated with indicated vaccine regimens. Mice immunized with indicated vaccines or appropriate controls, DNA plasmid with no insert (DNA control) and replicon expressing influenza HA (replicon control) (**A**) **to** (**C**) were challenged with 1000 PFU of RVFV ZH501 and monitored for clinical signs associated with RVFV infection and mortality daily post-challenge. (**D**) Mice were evaluated daily and scored for individual symptoms. Ruffled fur (absent = 0, present = 1), activity (normal = 0, reduced = 1), hunched (absent = 0, present = 1). The final score was the addition of each individual score. The minimum score was 0 for healthy and 1–3 depending upon the severity. A two-way ANOVA with Bonferroni's post tests was used to determine the significance of sickness score data between different groups, which is denoted by asterisks; **P<0.01, *** P<0.001.

The mice were challenged two weeks after final vaccination with a lethal dose (1×10^3^ PFU) ofRVFV ZH501. All the mice vaccinated with an all Gn-C3d-DNA or Rep-Gn strategy or in a DNA prime/replicon boost strategy were protected from virulent virus challenge with no body weight loss or development of clinical signs (Fig. 6, 7, and Table 3). Sixty percent of mice that received Gn without the molecular adjuvant C3d displayed ruffled fur and lethargy with one mouse succumbing to infection (Table 3 and Fig. 6D). As expected, all the mice immunized with MP12 and then challenged with RVFV ZH501 survived lethal challenge with no clinical signs of infection (Table 3 and Fig. 6D). However, mice vaccinated with WIV MP12 were not protected from challenge with all mice exhibiting reduced body weight (Fig. 6C), ruffled fur, lethargy (Table 3), and all mice ultimately succumbing to infection (Fig. 6D). Unvaccinated naive mice had severe signs of infection and body weight loss which resulted in all mice succumbing to infection by day 4 post-challenge (Fig. 6D). Mice that received appropriate DNA and replicon controls displayed clinical signs of infection (Fig 7A and B) and mortality was also observed in the control groups.

**Table 3:** Clinical profile of mice vaccinated with indicated vaccines or controls.

GROUPS	RUFFLED FUR	LETHARGY	HUNCHED	PARALYTIC SIGNS	Hemorrhage	DEAD/EUTHANIZED
Gn	3/5	3/5	0/5	0/5	0/5	1/5
Gn-C3d	0/5	0/5	0/5	0/5	0/5	0/5
Rep-Gn	0/5	0/5	0/5	0/5	0/5	0/5
Rep-Gn.wk6	5/5	5/5	1/5	0/5	0/5	3/5
DNA-C3d/Rep-Gn	0/5	0/5	0/5	0/5	0/5	0/5
MP12.wk0	0/5	0/5	0/5	0/5	0/5	0/5
MP12.wk6	0/5	0/5	0/5	0/5	0/5	0/5
WIV MP12	4/5	4/5	1/5	1/5	0/5	5/5
DNA control	5/5	5/5	0/5	0/5	0/5	2/5
Rep control	5/5	5/5	0/5	1/5	0/5	4/5
Mock vaccinated	5/5	5/5	0/5	0/5	0/5	5/5

Numbers in the parentheses indicate the number of immunizations performed for each vaccine or control.

### Passive sera transfer protects mice from virus infection

Pooled antiserum from each vaccinated group was transferred (i.p.) into unimmunized mice, which were then challenged with a lethal dose of RVFV ZH501 (Table 4). Eighty percent of mice that received sera from MP12 immunized mice survived challenge. A similar outcome was observed in the Gn-C3d group where 80% of mice survived. Sera from mice primed with Gn-C3d-DNA and then boosted with Rep-Gn or immunized with Rep-Gn protected 40% (2/5) of mice, which was similar to the mice that received sera from Gn-DNA vaccinated mice. All the mice that received sera from WIV MP12 immunized mice or mice that received sera from control immunized mice (DNA control, Rep control, Naïve) succumbed to virulent RVFV ZH501 infection.

**Table 4:** Passive transfer of sera from vaccinated mice protects against lethal RVFV challenge.

Vaccine Groups	Survivors
Gn	3/5
Gn-C3d	4/5
Rep-Gn	2/5
Gn-C3d/Rep-Gn	2/5
MP12.wk0	4/5
MP12.wk6	5/5
WIV MP12	0/5
DNA Control	0/5
Rep Control	0/5
Naive	0/5
Mock challenged	5/5

Five to six weeks old BALB/c mice were pre-treated with 100 µl of 1∶10 diluted serum from the indicated vaccinated mice or naïve animals by intraperitoneal injection. One hour post-sera inoculation mice were infected with 1000 PFU of RVFV by intraperitoneal route and monitored for survival.

## Discussion

One of the goals of an effective RVFV vaccine is to elicit protective neutralizing antibodies. In recent years, several RVFV vaccines strategies have been employed to elicit a potent neutralizing antibody responses [36], [38], [39], [40], [41], [42], [43], however, these vaccines did not always elicit high titer immune responses that protected against lethal challenge. In addition, several of these innovative strategies may not be appropriate for human use. Early RVFV vaccine studies focused on live-attenuated and inactivated virus strategies that induce long-lasting protection [36], [44], [45]. However, the induction of adverse reactions may likely limit the wide spread use of live-attenuated vaccines [15], [16], [18]. In contrast, inactivated virus vaccines often require multiple immunizations to elicit protective immune responses [46] and there is concern that the immunity elicited by these vaccines may rapidly wane without frequent booster vaccinations. In addition to potential concerns about safety or efficacy, due to the bioterrorism potential, ability to create virgin soil epidemics and zoonotic importance of RVFV, sero-surveillance is of major importance in the international trade of animals and animal-related products. Marker vaccines make it possible to differentiate infected from vaccinated animals [47]. Diagnostic tests such as RVFV recombinant N protein based ELISA and immuno-fluorescence performed on infected or transfected cells or tissues are widely used in laboratories for RVFV diagnosis [48], [49], [50], [51]. Therefore, an ideal vaccine, especially for livestock applications, would lack the RVFV N protein, which would allow differentiation between vaccinated and infected individuals.

To overcome the limitations discussed above, we have developed two promising vaccine candidates based on DNA plasmid and alphavirus replicon vectors that express the virus envelope glycoprotein, Gn. Each vaccine was tested alone or in a DNA prime/replicon boost strategy formulation to elicit protective immune response against virulent RVFV infection in mice. DNA vaccines have been licensed for veterinary use [52]. However, DNA vaccines have been less effective in human clinical trials for other infectious diseases [52], [53]. In order to enhance the antibody responses elicited by DNA vaccines, our laboratory has pioneered the use of the complement protein C3d as a molecular adjuvant [20], [23], [25]. Since the Gn glycoprotein is known to contain protective neutralizing epitopes [38], [40], [42], [54], we focused our efforts on characterizing whether fusion of the C3d molecule to Gn resulted in enhanced RVFV specific immunity. Mice vaccinated with Gn-C3d-DNA had high titer neutralizing antibodies compared to mice vaccinated with DNA expressing Gn alone. It remains to be determined whether this effect is solely due to C3d's function as a molecular adjuvant or whether the fusion of C3d also enhances the secretion of Gn from the cell or the protein's stability in the extracellular environment.

In addition to the DNA vaccine strategy, we also used a DNA prime/alphavirus replicon boost strategy to expand the repertoire of elicited immune responses. Previously, our group used a Sindbis virus replicon vectors expressing the RVFV Gn and Gc glycoproteins, as well as the non-structural NsM protein to induce protective immune responses in mice against RVFV [29]. In this study, the replicons administered alone or in a DNA prime/replicon boost strategy elicited similar anti-Gn antibody titers, however, different subclasses of IgG were elicited by each vaccine. The isotype of the polyclonal antibody in part determines the effector functions of the anti-Gn antibodies and identifies the T helper cell bias (required for antibody class switching). The predominant isotype elicited by DNA and replicon immunizations was IgG1 indicating a Th2 bias. Antibodies of the IgG2a/c and IgG2b subclass fix complement proteins C1q and C3 and can opsonize and inhibit infection. IgG2a/c binds FcγRI with high avidity facilitating enhanced uptake of virus-antibody complexes by macrophages. The predominant IgG isotype elicited by DNA vaccination was IgG1 indicating a Th2 bias. However, IgG1, IgG2a, and IgG2b were detected in both replicon vaccinated, as well as live MP12 immunized mice (Fig. 3), indicating that both the replicon and the live attenuated vaccine elicit a mixed T-helper response.

Even though both MP12 infection and the WIV vaccination elicited the highest anti-Gn titers, only the live MP12 infection elicited strong neutralizing antibody responses (**Fig. 4**). Gn-C3d-DNA and Gn-C3d-DNA/Rep-Gn vaccinated mice had statistically similar neutralizing titers as MP12 immunized mice. Studies in the past have mainly focused on survival of vaccinated mice post-challenge; however an ideal vaccine should not only be able to protect from virus infection, but also prevent development of clinical symptoms. In this study, we evaluated our candidate vaccines for the ability to confer protection, as well as ability to prevent clinical signs. Few mice from DNA and replicon control groups survived virus infection similar to previous studies [38], [39], however all of the control mice displayed clinical signs of infection that was characterized by ruffled fur and lethargy. We observed a correlation between neutralizing antibody titers and development of clinical signs or mortality. Mice with a PRNT_50_ value of <1∶10 succumbed to lethal infection and a PRNT_50_ value of ≥1∶40 was sufficient to prevent clinical signs. This indicates that the clinical sickness score more accurately reflected vaccine efficacy in preventing RVFV infection and may be a useful tool for future vaccine studies.

The issue of survival from control DNA or mock vaccination is curious, but has been observed in previous publications. Spik et al. [39] also saw survival of a subset of mice following vaccination with DNA controls up to 31 days following challenge with Rift Valley fever virus. In addition, Bird et al. observed that sham mice did not succumb to lethal Rift Valley fever virus challenge, but they developed severe clinical signs of ruffled fur, hunched back, and lethargy [48].

To further explore the ability of factors in the sera to protection mice from RVFV infection, passive transfer of serum from vaccinated mice to naïve mice demonstrated that humoral immune response play a major role in anti-RVFV immunity (Table 4) [40]. Not all mice passively administered the serum were protected, which may be due to dilution of the neutralizing antibodies during preparation that resulted in lack of protection in some mice. Mice vaccinated with replicons alone or in a DNA prime/replicon boost strategy, but not by DNA alone, had robust cellular responses directed at Gn. Cellular responses are critical for clearing virally infected cells in many systems. Although the elicitation of robust neutralizing antibodies are considered ideal for the development of an effective RVFV vaccine, induction of cellular responses by immunization may clear virally infected cells, reduce morbidity, and hasten recovery from infection. The replicon-based vaccines elicited cellular immune responses against the Gn protein, but Gn expressed from DNA plasmids did not, even though priming mice with DNA did not dampen the induction of cellular responses by the Gn-C3d/Rep-Gn in the DNA prime/replicon boost regimen (Table 2). Although non-specific induction of T-cell responses against RVFV glycoproteins and nucleocapsid proteins have been previously reported [55], this is the first report to identify an MHC-I restricted immunodominant epitope (SYAHHRTLL) on the surface of Gn as predicted by mutlitple algorithm methods to detect the peptide sequence with lowest IC50 and hence better binding to MHC-I [www.immuneepitope.org]. Although, both Rep-Gn alone or in a DNA prime/replicon boost approach Gn-C3d/Rep-Gn induced a combination of humoral and cell mediated immune responses and therefore this strategy may warrant further evaluation in large animals and humans.
